# Testicular Sertoli Cell Membrane-Modified Schisandrin A-Loaded Catalase Nanoparticles Ameliorate Cytoxan-Induced Testicular Injury

**DOI:** 10.34133/bmr.0273

**Published:** 2025-11-04

**Authors:** Yisong Ju, Li Lu, Jihai Liu, Lei Qian, Haoqiang Zhang, Fuying Zhu, Yuhang Li, Xun Wang, Tao Song, Qingsong Ye, Ao Ma, Xiaozhi Zhao

**Affiliations:** ^1^ Department of Andrology, Nanjing Drum Tower Hospital Clinical College of Nanjing University of Chinese Medicine, Nanjing, Jiangsu 210008, China.; ^2^ Department of Andrology, Nanjing Drum Tower Hospital, the Affiliated Hospital of Nanjing University Medical School, Nanjing, Jiangsu 210008, China.; ^3^ Department of Andrology, Nanjing Drum Tower Hospital Clinical College of Nanjing Medical University, Nanjing, Jiangsu 210008, China.; ^4^ State Key Laboratory of Pharmaceutical Biotechnology, School of Life Sciences, Nanjing University, Nanjing, Jiangsu 210093, China.

## Abstract

Cytoxan (CTX), an alkylating chemotherapeutic agent, produces severe testicular toxicity in male patients by generating mitochondrial dysfunction and redox imbalance, resulting in an excess buildup of reactive oxygen species (ROS) that compromises sperm function and fertility. Schisandrin A (SchA), a bioactive compound derived from traditional Chinese medicine *Schisandra chinensis*, can alleviate this injury by mimicking superoxide dismutase activity to scavenge ROS, but its poor water solubility, inability to penetrate the blood–testis barrier (BTB), and high dosage requirement that cause hepatic and gastrointestinal side effects limit clinical application. This study describes a biomimetic SchA delivery system that leverages TM4 (Sertoli-like) cell membrane-coated protein carriers to enable some degree of targeted delivery to testicular tissue. We named it CAT-SchA@SCM. Briefly, SchA was loaded onto catalase (CAT), and protein nanoparticles were disguised with TM4 cell membranes. Transmission electron microscopy and dynamic light scattering results showed that CAT-SchA@SCM had an average particle size of around 170 nm and was well-dispersed and stable. In vitro and in vivo experiments demonstrated that CAT-SchA@SCM could target Sertoli cells to a limited extent, cross the BTB, considerably scavenge intracellular ROS, inhibit apoptosis, and effectively protect Sertoli cells. In a mouse model of CTX-induced testicular damage, CAT-SchA@SCM dramatically increased sperm motility, testicular weight, and serum testosterone levels. Hematoxylin and eosin staining of major organs demonstrated that CAT-SchA@SCM exhibited favorable biosafety. Additionally, activation of the Nrf2–HO-1 signaling pathway was observed, indicating a potential mechanism underlying the therapeutic effect of CAT-SchA@SCM. Therefore, the findings of this study suggest that this nanocomplex-based treatment may offer a viable approach for addressing CTX-induced testicular injury, with potential clinical applications in the future.

## Introduction

Cytoxan (CTX), an alkylating antineoplastic drug, is widely used in clinical practice and plays a important role in treating leukemia, lymphoma, and various solid tumors [1–3]. However, its severe testicular toxicity significantly limits clinical application, threatening the long-term quality of life of young male cancer patients [2,4]. Most male patients experience spermatogenic dysfunction after CTX treatment, including a reduction in the volume of the testicles, and impaired sperm motility [5,6]. The main reason for the testicular injury caused by CTX is that during its metabolic process, it will produce metabolites that interfere with the function of mitochondria, and it will inhibit the activity of antioxidant enzymes in the metabolic process, thus leading to an imbalance in the redox reaction [5,7,8]. Excessive reactive oxygen species (ROS) accumulate, especially superoxide anions (O^2−^) and hydrogen peroxide (H_2_O_2_), which damage the membrane structure and DNA integrity of sperm, and thus affect the function and survival rate of sperm [7,9].

Previous studies have shown that schisandrin A (SchA), a compound abundant in the traditional Chinese herb *Schisandra chinensis*, can alleviate testicular injury to a certain extent by mimicking the biological functions of superoxide dismutase, scavenging ROS, and reducing oxidative damage [10]. However, its poor water solubility, low bioavailability, and inability to penetrate the blood–testis barrier (BTB) necessitate high dosages to achieve the desired protective effects [11,12]. This, in turn, leads to severe side effects such as increased hepatic burden and gastrointestinal reactions, significantly limiting its clinical application [13,14]. In summary, the primary challenge in using SchA to ameliorate CTX-induced testicular injury lies in achieving testicular-targeted delivery while minimizing adverse effects on healthy cells [15–17].

Nanocarriers such as proteins offer an effective drug delivery strategy due to their high drug-loading capacity, sustained-release properties, and a certain degree of biocompatibility [18]. However, they also face challenges, such as rapid clearance by immune cells and the lack of targeting to testicular tissue [19,20]. Membrane-coated nanomaterials provide a new approach to overcome these challenges [21]. Testicular Sertoli cell membranes (SCMs), which are the main constituent cells of the BTB, endow the coated nanomaterials with major histocompatibility, enabling them to evade rapid clearance by immune cells [22]. Meanwhile, the expression of chemokines and adhesion molecules on the membrane can endow the nanomaterials with a certain degree of targeting to Sertoli cells [23]. These unique biological properties hold great potential for addressing challenges related to protein drug delivery [22,24,25].

**Fig. 1.**
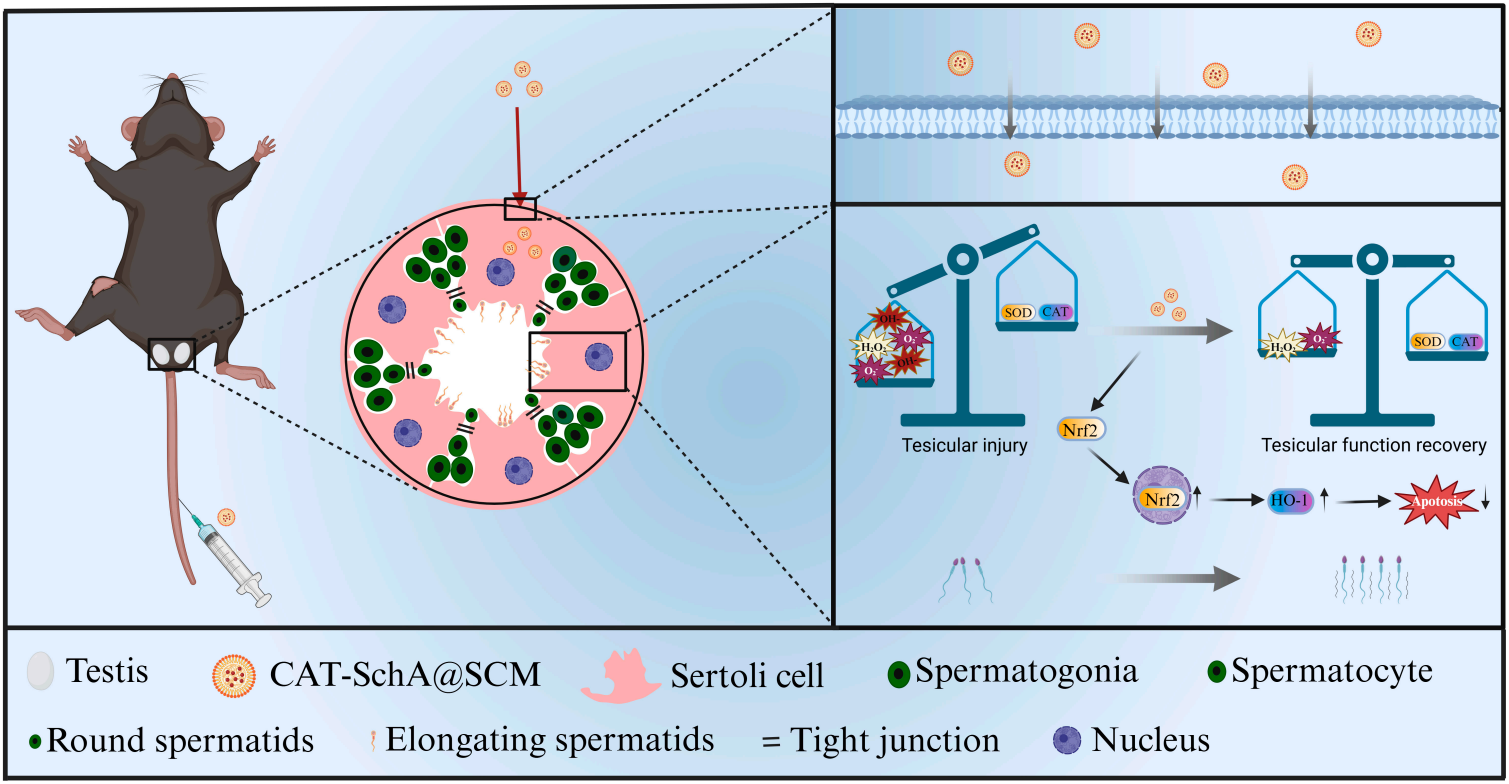
The mechanism of CAT-SchA@SCM in alleviating cytoxan-induced testicular injury by targeting Sertoli cells.

In this study, we found that the TM4 (Sertoli-like) cell membrane can target Sertoli cells and have a certain ability to cross the BTB. In a mouse model of testicular injury induced by CTX, we found that CAT-SchA@SCM can significantly improve the sperm motility, testes weight, testis-to-body weight ratio, and other indicators of the mice, and it does not exhibit obvious biological toxicity. Therefore, our results suggest that CAT-SchA@SCM has the potential to treat testicular injury caused by CTX (Fig. 1).

## Materials and Methods

### Materials

SchA, CAT, DiD (1,1'-dioctadecyl-3,3,3',3'-tetramethylindodicarbocyanine, 4-chlorobenzenesulfonate salt) fluorescent dye, 4′,6-diamidino-2-phenylindole (DAPI), radioimmunoprecipitation assay lysis buffer, Cell Counting Kit-8 (CCK-8) detection kit, and bicinchoninic acid (BCA) protein detection kit were purchased from Beyotime Biotechnology. CTX was purchased from Endoxan. 4-Hydroxycyclophosphamide (4-HC) was purchased from MCE. Phosphatase inhibitors and protease inhibitors were provided by BosterBio. Creatinine (CREA), blood urea nitrogen (BUN), alanine aminotransferase (ALT), aspartate aminotransferase (AST) detection kits, and Live/Dead Stain kits were purchased from Elabscience. Hematoxylin and eosin (H&E) staining reagents were obtained from Servicebio. Dulbecco’s modified Eagle’s medium (DMEM) and F12 media were purchased from Vicente. Fetal bovine serum (FBS) was provided by Gemini. Penicillin–streptomycin was purchased from Gibco.

### Cell culture

TM3 and TM4 cells were cultured in a medium containing 90% (v/v) F12, 10% (v/v) FBS, and 1% penicillin [10 kilo units (KU)/ml]/streptomycin (10 mg/ml) at 37 °C in a 5% CO₂ environment. GC1 cells were cultured in a medium containing 90% (v/v) DMEM, 10% (v/v) FBS, and 1% penicillin (10 KU/ml)/streptomycin (10 mg/ml) at 37 °C in a 5% CO₂ environment.

### Animal research

C57BL/6 mice [6 to 8 weeks old, 22 to 25 g, male, specific pathogen-free (SPF)] were provided by Huachuang Xinnuo Chinese Medicine Technology Co. Ltd. (Jiangsu, China). All animal experiments were carried out in accordance with the Experimental Animal Ethics Guidelines of Nanjing Drum Tower Hospital and approved by the Ethics Committee of Nanjing Drum Tower Hospital (2021AE01035).

A mouse model of testicular injury induced by CTX (50 mg/kg, intraperitoneal injection, for 7 consecutive days) was established. In total, it is divided into 5 groups, namely, the normal saline group and the CTX group. The treatment groups are divided into 3 groups: the group receiving oral administration of SchA, the group receiving tail vein injection of CAT-SchA, and the group receiving tail vein injection of CAT-SchA@SCM. After continuous administration for 7 d, the testis and epididymal tail tissues of the mice were taken out and the sperm parameters were analyzed to evaluate the therapeutic effect.

### Synthesis and characterization of CAT-SchA@SCM

CAT enzyme (5 mg) was weighed, dissolved in 5 ml of double-distilled water (ddH₂O), and stirred at room temperature; 100 μl of SchA (dissolved in CHCl₃) at a concentration of 100 mg/ml was slowly added; and then 200 μl of TM4 cell membrane suspension (1 mg/ml) was added. A cell disruptor was used to perform ultrasonic treatment under ice-bath conditions (55% intensity, 3 s of sonication, 4 s of pause, for 4 min) to prepare CAT-SchA@SCM nanoparticles. Then, CHCl₃ was blow-dried with a gas cylinder to obtain CAT-SchA@SCM nanoparticles.

### Extraction of TM4 cell membrane

TM4 cells were collected in a 10-cm culture dish and resuspended in 1 ml of 0.25× phosphate-buffered saline (PBS). The cell suspension was freeze-thawed 3 times between liquid nitrogen and room temperature to break the cells and centrifuged at 700*g* for 5 min at 4 °C to remove large organelles. The supernatant was taken and centrifuged at 14,000*g* for 10 min at 4 °C. The collected precipitate is the cell membrane.

### In vitro uptake of CAT-SchA@SCM by TM4 cells

CAT-SchA@SCM nanoparticles were labeled with DiD fluorescent dye. The labeled nanoparticles were added to the wells with TM4 cell coverslips at different time points (1, 2, and 3 h). After the set time, the medium was discarded, washed 3 times with PBS, and fixed with 4% paraformaldehyde at room temperature for 15 min. The coverslips were mounted with an anti-quenching mounting medium containing DAPI. The uptake of nanoparticles was observed under a confocal microscope.

### In vitro treatment effects of CAT-SchA@SCM on 4-HC-induced cells

TM3, TM4, and GC1 cells were firstly incubated with 4-HC for 24 h, and then SchA, CAT-SchA, and CAT-SchA@SCM were added at the same concentration of SchA. The CCK-8 method was used to detect cell viability 24 h after CAT-SchA@SCM treatment.

### In vivo distribution of CAT-SchA@SCM in normal mice

CAT-SchA@SCM was injected into normal mice. The main organs such as the heart, liver, spleen, lung, kidney, and testis were collected at time points of 0, 2, 4, 8, 12, and 24 h. A small animal imaging instrument was used to collect fluorescence signals, and the mean fluorescence intensity (MFI) of each organ was analyzed.

### Cytotoxicity assay of CAT-SchA@SCM

The cytotoxicity of SchA, CAT-SchA, and CAT-SchA@SCM on TM3, TM4, and GC1 cells was evaluated by the CCK-8 method. Cells were inoculated at a density of 1 × 10^4^ cells per well. After the cells have grown for 24 h, the medium was discarded and then drugs of different concentrations were added and incubation was continued for another 24 h. The CCK-8 kit was used to detect cell viability and calculate the cell survival rate.

### Sperm motility analysis

The mice were euthanized, and the cauda epididymis was collected, cut into pieces, and incubated in 800 μl of DMEM containing 10% FBS prewarmed at 37 °C for 5 min to release sperm. The sample was gently mixed, and then a computer-assisted sperm analysis system (CASA) was used to analyze sperm motility parameters, including the total number of sperm and the proportion of motile sperm.

### Statistical analyses

GraphPad Prism 10 was used for statistical analysis. Data are presented as mean ± standard error of the mean (SEM). Student’s *t* test was applied for pairwise comparisons, while one-way analysis of variance (ANOVA) with the Newman–Keuls multiple comparison test was used for multiple group comparisons. A *P* value of <0.05 was considered statistically significant.

## Results

### Preparation and characterization of CAT-SchA@SCM

**Fig. 2.**
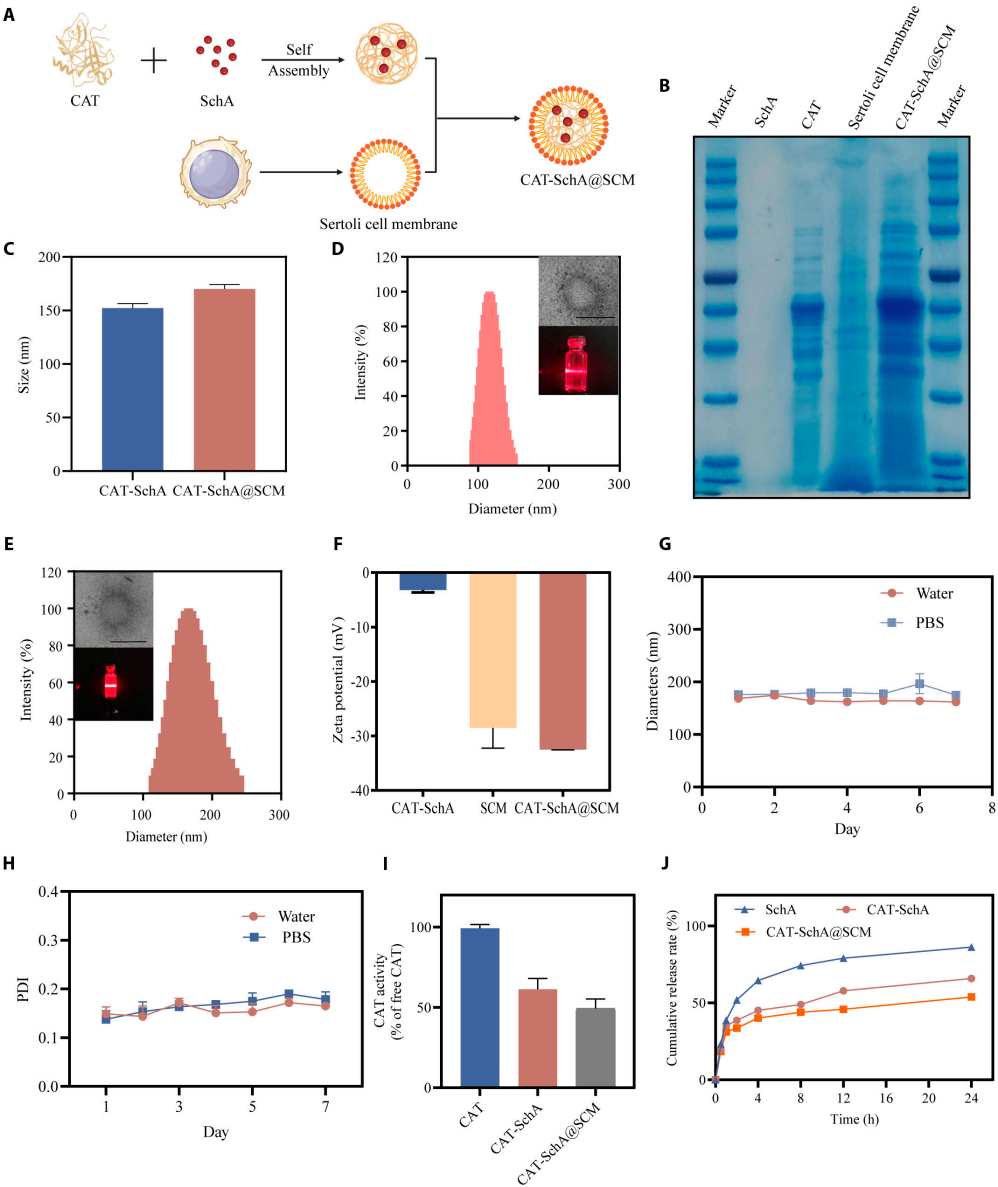
Preparation and characterization of CAT-SchA@SCM. (A) Preparation process of CAT-SchA@SCM. (B) SDS-PAGE analysis of SchA, CAT, TM4-CM, and CAT-SchA@SCM to confirm the successful encapsulation of CAT-SchA by TM4-CM. (C) The average particle sizes of the synthesized CAT-SchA and CAT-SchA@SCM were measured by DLS (*n* = 3). (D) Particle size distribution of CAT-SchA measured by DLS and TEM. Scale bar, 200 nm. (E) Particle size distribution of CAT-SchA@SCM measured by DLS and TEM. Scale bar, 200 nm. (F) Zeta potentials of CAT-SchA, TM4 cell membrane, and CAT-SchA@SCM. (G) Stability of CAT-SchA@SCM in aqueous solution and PBS for 7 d (*n* = 3). (H) Polymer dispersity index changes of CAT-SchA@SCM in pure water and PBS. (I) Detection of CAT enzyme activity for CAT-SchA and CAT-SchA@SCM (*n* = 3). (J) SchA in vitro release from SchA solution, CAT-SchA, and CAT-SchA@SCM after incubation in water (*n* = 3). Data were presented as mean ± SEM.

In brief, for the preparation process (Fig. 2A), first, SchA and CAT enzyme form a CAT-SchA nanonuclear through protein self-assembly and hydrophobic interaction. Subsequently, the TM4 cell membrane is coated on the surface of the CAT-SchA nanonuclear by the ultrasonic emulsification method, and finally, the CAT-SchA@SCM nanoparticles are synthesized. To verify whether the nanoparticles were successfully synthesized, we analyzed the protein composition of CAT-SchA@SCM by sodium dodecyl sulfate–polyacrylamide gel electrophoresis (SDS-PAGE) (Fig. 2B). The results showed that the characteristic protein bands of both CAT enzyme and TM4 cell membrane were present in CAT-SchA@SCM, confirming that TM4-CM had successfully encapsulated the CAT-SchA nanocore. Subsequently, the average particle size of the nanocore measured by the dynamic light scattering (DLS) method is about 152 nm. The CAT-SchA@SCM particle size measured by the DLS method is about 170 nm (Fig. 2C). DLS and transmission electron microscopy (TEM) characterization further verified the subspherical morphology and homogeneous distribution of both CAT-SchA and CAT-SchA@SCM (Fig. 2D and E). The zeta potential of CAT-SchA@SCM is −28.5 mV (Fig. 2F). The relatively high negative charge value indicated a large surface charge density, which could effectively prevent particle aggregation through electrostatic repulsion, thus exhibiting good colloidal stability and dispersibility.

To further evaluate its application potential, we investigated the stability of CAT-SchA@SCM in different media. It was dispersed in pure water and PBS solution, and its particle size changes and polymer dispersity index (PDI) were observed at different time points. The results showed that CAT-SchA@SCM could remain stable for a long time in both media without significant aggregation or degradation (Fig. 2G and H). This excellent stability ensured the performance of the nanoparticles during storage, transportation, and blood circulation, thereby improving the drug delivery efficiency. Furthermore, we used a CAT assay kit to detect the ability of free CAT enzyme, CAT-SchA, and CAT-SchA@SCM to scavenge H₂O₂. It was found that after the CAT enzyme was modified with SCM, it could still retain approximately 50% of its activity (Fig. 2I). That is to say, CAT-SchA@SCM can effectively maintain the activity of CAT enzyme, which is conducive to subsequent biological applications. We hypothesize that the reason for the decreased activity of CAT-SchA and CAT-SchA@SCM compared to CAT enzyme lies in the sustained-release effect of nanoparticles and the incomplete encapsulation of CAT-SchA by SCM. Finally, we calculated the release degrees of SchA in SchA solution, CAT-SchA, and CAT-SchA@SCM in aqueous solution using the dialysis method (Fig. 2J). We found that 60% of SchA was released from the SchA solution within the first 4 h; however, CAT-SchA and CAT-SchA@SCM exhibited better sustained-release properties within the first 24 h.

### CAT-SchA@SCM targets Sertoli cells

**Fig. 3.**
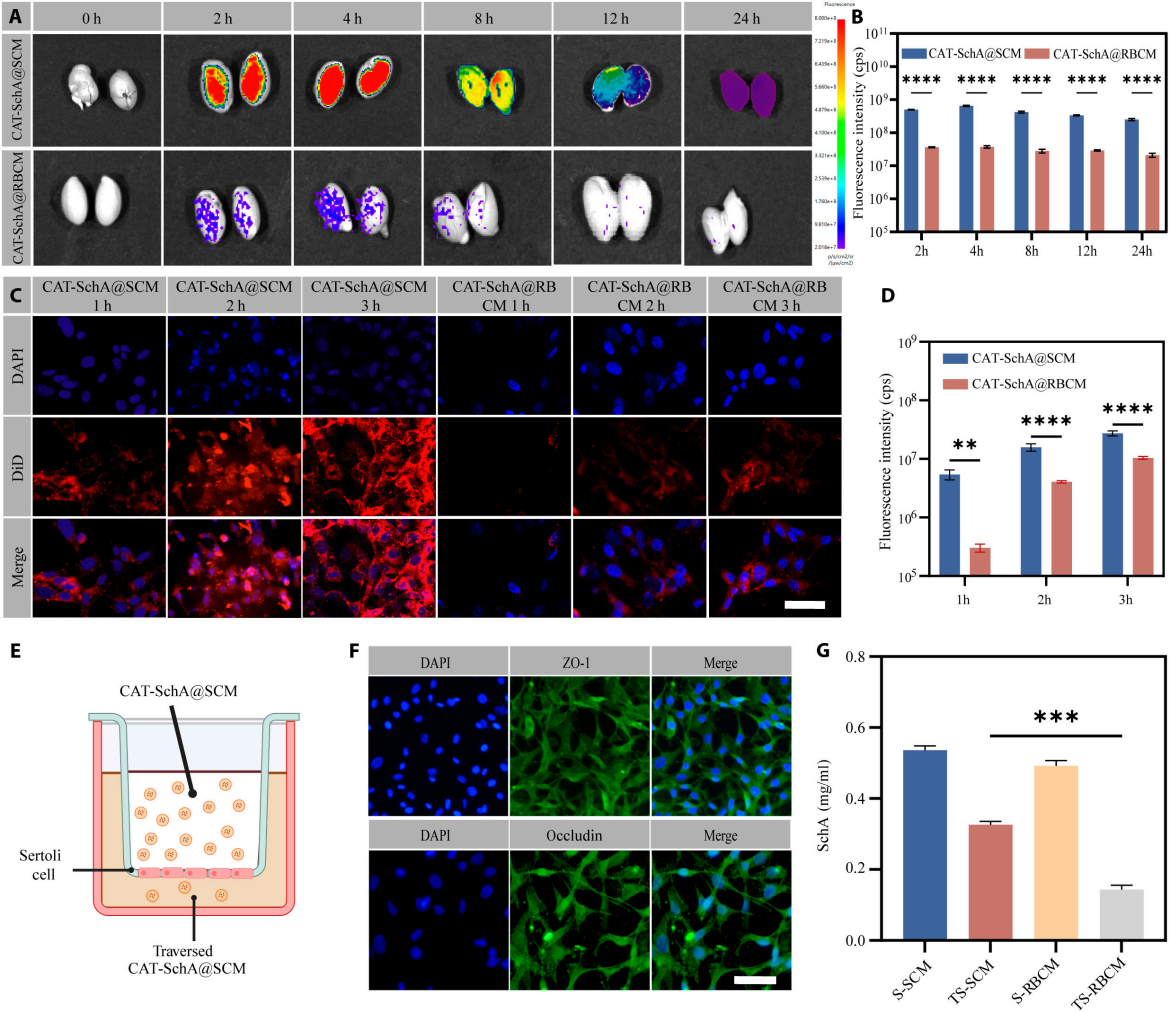
CAT-SchA@SCM specifically accumulates in the Sertoli cells and can cross the BTB to a certain extent. (A) In vivo imaging of the distribution of DiD-labeled CAT-SchA@SCM and CAT-SchA@RBCM in the testes of healthy mice at different time points. (B) Region of interest (ROI) signal analysis of ex vivo testis tissue imaging at different time points (*n* = 3). (C) Laser confocal microscopy images of TM4 cells co-incubated with DiD-labeled CAT-SchA@SCM and CAT-SchA@RBCM at an SchA concentration of 1 mg/ml for 1, 2, and 3 h. The nuclei of TM4 cells were stained with DAPI. Scale bar, 50 μm. (D) Quantitative analysis of the average fluorescence intensity of DiD-labeled CAT-SchA@SCM and CAT-SchA@RBCM co-incubated with TM4 cells for 1, 2, and 3 h (*n* = 3). (E) Schematic illustration of CAT-SchA@SCM crossing the in vitro BTB model. (F) Expression of tight junction proteins (ZO-1 and occludin) in Sertoli cells. Scale bar, 50 μm. (G) The concentration of SchA was detected by HPLC (*n* = 3). Data were presented as mean ± SEM. Statistical differences between 2 groups were analyzed by using 2-tailed Student’s *t* test. **P < 0.01, ***P < 0.001, ****P < 0.0001.

To investigate whether CAT-SchA@SCM could specifically target testicular tissue, we prepared CAT-SchA@RBCM with the red blood cell membrane for comparison (Fig. S1A to D). First, we injected mice with DiD-labeled CAT-SchA@SCM and CAT-SchA@RBCM via the tail vein. Subsequently, the animals were euthanized at multiple time points post-injection (2, 4, 8, 12, and 24 h), and major organs including the heart, liver, spleen, lungs, kidneys, and testes were harvested for ex vivo fluorescence imaging analysis. The results showed that the fluorescence intensity of testes in the CAT-SchA@SCM group was consistently higher than that in the CAT-SchA@RBCM group from 2 to 24 h after injection (Fig. 3A and B), indicating the better accumulation of CAT-SchA@SCM in testes.

To further investigate whether CAT-SchA@SCM could specifically target Sertoli cells, DiD-labeled CAT-SchA@SCM and CAT-SchA@RBCM were co-incubated with TM4 cells, and the uptake behavior of the cells was observed at 1, 2, and 3 h of co-incubation (Fig. 3C and D). The results showed that with the extension of time, the uptake amount of nanoparticles by the cells increased significantly, and the uptake efficiency of TM4 cells for CAT-SchA@SCM was much higher than that for CAT-SchA@RBCM. Therefore, we conclude that CAT-SchA@SCM can be efficiently taken up by TM4 cells, and the uptake efficiency increases with time.

In addition, the ability of CAT-SchA@SCM to cross the BTB was studied. An in vitro BTB model was constructed by culturing TM4 cells on cell slides and simultaneously in Transwell chambers (Fig. 3E). Immunofluorescence (IF) staining showed the expression of tight junction proteins occludin and ZO-1 in TM4 cells, indicating the formation of tight junctions and successful construction of the BTB model (Fig. 3F). Subsequently, CAT-SchA@SCM and CAT-SchA@RBCM were added to the medium in the upper chamber and incubated for 24 h. After incubation, the media were collected and the concentration of SchA was determined by high-performance liquid chromatography (HPLC) (Fig. 3G). The results showed that the concentration of SchA in the medium of the lower chamber was significantly higher than that of the CAT-SchA@RBCM group but lower than that of the medium in the upper chamber, indicating that CAT-SchA@SCM can cross the BTB.

### CAT-SchA@SCM alleviates 4-HC-induced cell damage by reducing ROS levels

**Fig. 4.**
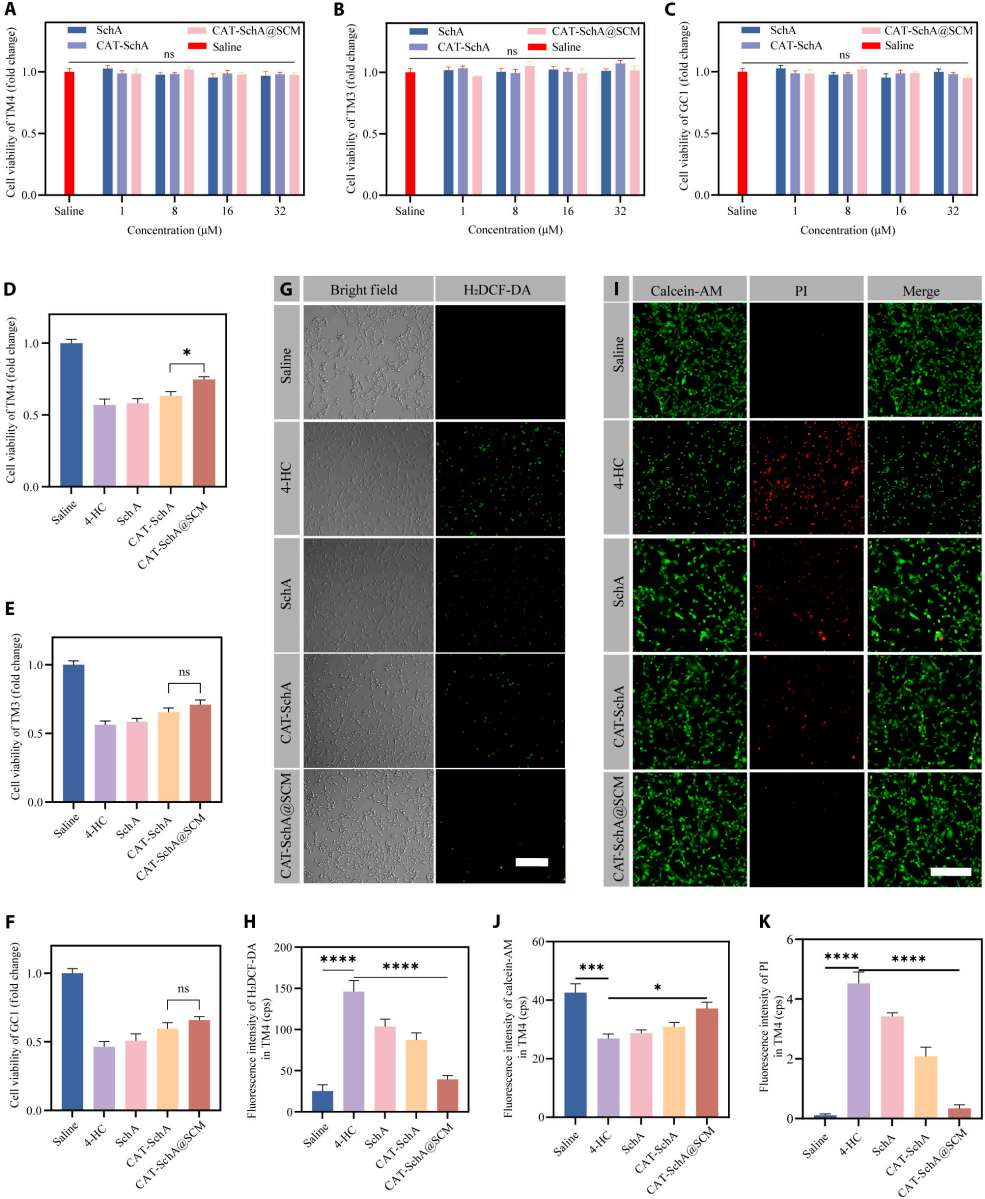
CAT-SchA@SCM improves TM4 cell apoptosis by reducing ROS levels. (A to C) Cytotoxicity of different concentrations of SchA, CAT-SchA, and CAT-SchA@SCM on TM4 (A), TM3 (B), and GC1 (C) cell lines (*n* = 6). (D to F) Recovery effect of different treatment groups on the cell viability of TM4 (D), TM3 (E), and GC1 (F) cell lines after 4-HC stimulation (*n* = 6). (G) Representative images of ROS in different treatment groups after 4-HC treatment (scale bar, 100 μm). (H) ROI signal quantification of ROS levels in each group of the TM4 cell line compared with the model group (*n* = 3). (I) Representative images of calcein-AM/propidium iodide (PI) in different treatment groups after 4-HC treatment. Scale bar, 100 μm. (J) ROI signal quantification of calcein-AM levels in each group of the TM4 cell line compared with the model group (*n* = 3). (K) ROI signal quantification of PI levels in each group of the TM4 cell line compared with the model group (*n* = 3). Data were presented as mean ± SEM. Statistical differences between 2 groups were analyzed by using 2-tailed Student’s *t* test. ns, no significant difference. *P < 0.05, ***P < 0.001, and ****P < 0.0001.

CTX needs to be metabolically activated by enzymes in the body to function. Based on this, 4-HC, the active ingredient of CTX, was selected to establish a testicular cell damage model. We used CCK-8 kit to explore the concentration of 4-HC that induced cell damage in TM4, TM3, and GC1 cell lines (Fig. S2A to C) , and then investigated the protective effects of SchA, CAT-SchA, and CAT-SchA@SCM on 4-HC-induced cell damage. First, we evaluated the cytotoxicity of drugs on TM4, TM3, and GC1 cell lines. Specifically, SchA, CAT-SchA, and CAT-SchA@SCM at different concentrations were dissolved in the medium and cell viability was detected after 24 h. It was found that the cell viability of each group did not change significantly (Fig. 4A to C). Next, each of the 3 cell lines was divided into 5 groups, labeled as Saline, 4-HC, SchA, CAT-SchA, and CAT-SchA@SCM. After different treatments, the CCK-8 assay was used to detect cell viability. The results showed that the viability of the 3 types of cells in the 4-HC treatment group was significantly reduced, and treatment with the SchA group, CAT-SchA group, and CAT-SchA@SCM group could partially rescue this situation. Notably, the cell viability of the CAT-SchA@SCM group in TM4 cells was significantly higher than that of the CAT-SchA group, while in TM3 and GC1 cell lines, there was no significant difference (Fig. 4D to F). These results indicated that SchA could partially rescue the testicular cell damage induced by 4-HC, and the uptake efficiency of CAT-SchA@SCM by TM4 cells was significantly higher than that by TM3 and GC1 cell lines, highlighting the stronger targeting of CAT-SchA@SCM to TM4 cells.

To further explore the mechanism of action of the nanoparticles, we verified their ability to scavenge intracellular ROS. TM4 cells were treated as described before, and the intracellular ROS level was detected. The results showed that, compared with the control group, the level of cellular ROS in the 4-HC treatment group increased significantly, and all 3 treatment groups could reduce the ROS level to a certain extent, suggesting the ROS scavenging ability of SchA. Significantly, the ROS level in the CAT-SchA@SCM group was significantly superior to that of the other treatment groups, indicating a better ability to scavenge ROS (Fig. 4G and H). In addition, the levels of live and dead cells were detected, and the results showed that CAT-SchA@SCM significantly reduced the apoptosis induced by 4-HC (Fig. 4I to K). This series of results further confirmed that CAT-SchA@SCM had a significant protective effect on TM4 cells by scavenging intracellular ROS and inhibiting cell apoptosis, providing a strong basis for its application in the treatment of testicular injury.

### CAT-SchA@SCM improves CTX-induced testicular injury by regulating the Nrf2 and HO-1 pathways

**Fig. 5.**
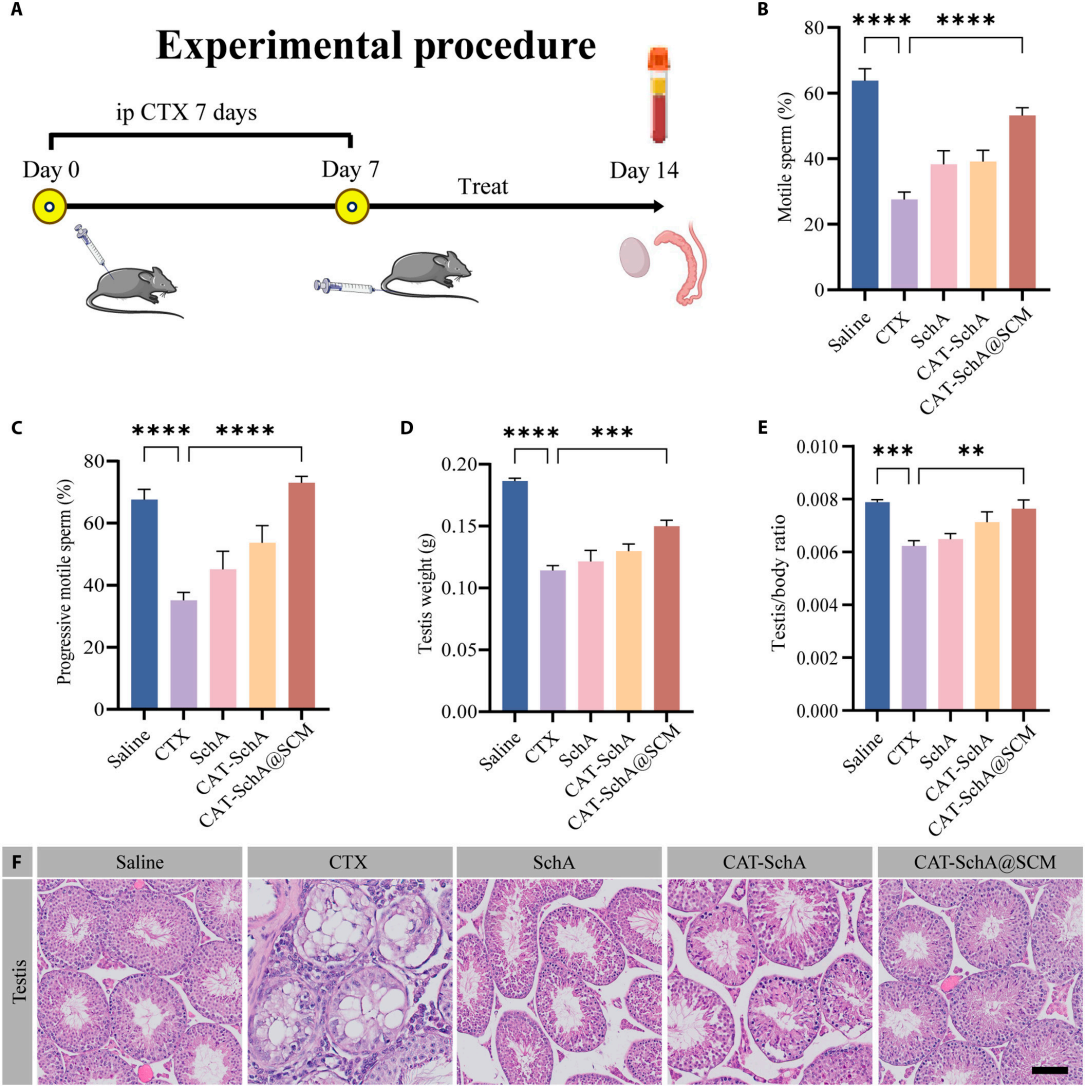
CAT-SchA@SCM improves the testicular injury caused by CTX and the decline in sperm quality. (A) Timeline and design of the animal experiment. After continuous intraperitoneal (ip) injection of CTX for 7 d, CAT-SchA@SCM was intravenously injected into the tail vein once every other day for 1 week, and the serum, testes, and epididymis of the mice were collected. (B to E) Detection of motile sperm (%) (B), progressive motile sperm (%), (C) testis weight (g) (D), and testes/body ratio (E) in testicular injury mice after different treatments (*n* = 6). (F) H&E staining of testicular tissues in different treatment groups. Scale bar, 100 μm. Data were presented as mean ± SEM. Statistical differences between 2 groups were analyzed by using 2-tailed Student’s *t* test. **P < 0.01, ***P < 0.001, and ****P < 0.0001.

To investigate the therapeutic effect of nanoparticles on testicular injury, we established a CTX-induced testicular injury model, and the drug administration schedule was as follows (Fig. 5A). Compared with the control group, main indicators of testicular function including sperm motility (Fig. 5B and C), testis weight (Fig. 5D), testis/body ratio (Fig. 5E), and serum testosterone level (Fig. S3) of mice in the model group decreased significantly. In the treatment groups, although the above indicators of the SchA group and the CAT-SchA group increased to a certain extent, there was no significant difference compared with the model group. However, when the mice were given the same dose, the CAT-SchA@SCM group showed obvious improvement in these indicators. In addition, H&E staining showed that the seminiferous epithelium of the testis in the model group became thinner and incomplete. The CAT-SchA group showed a greater number of recovered spermatogenic cells and a more remarkable therapeutic effect than did the SchA group, while the CAT-SchA@SCM group outperformed both the SchA and CAT-SchA groups in these 2 aspects (Fig. 5F).

**Fig. 6.**
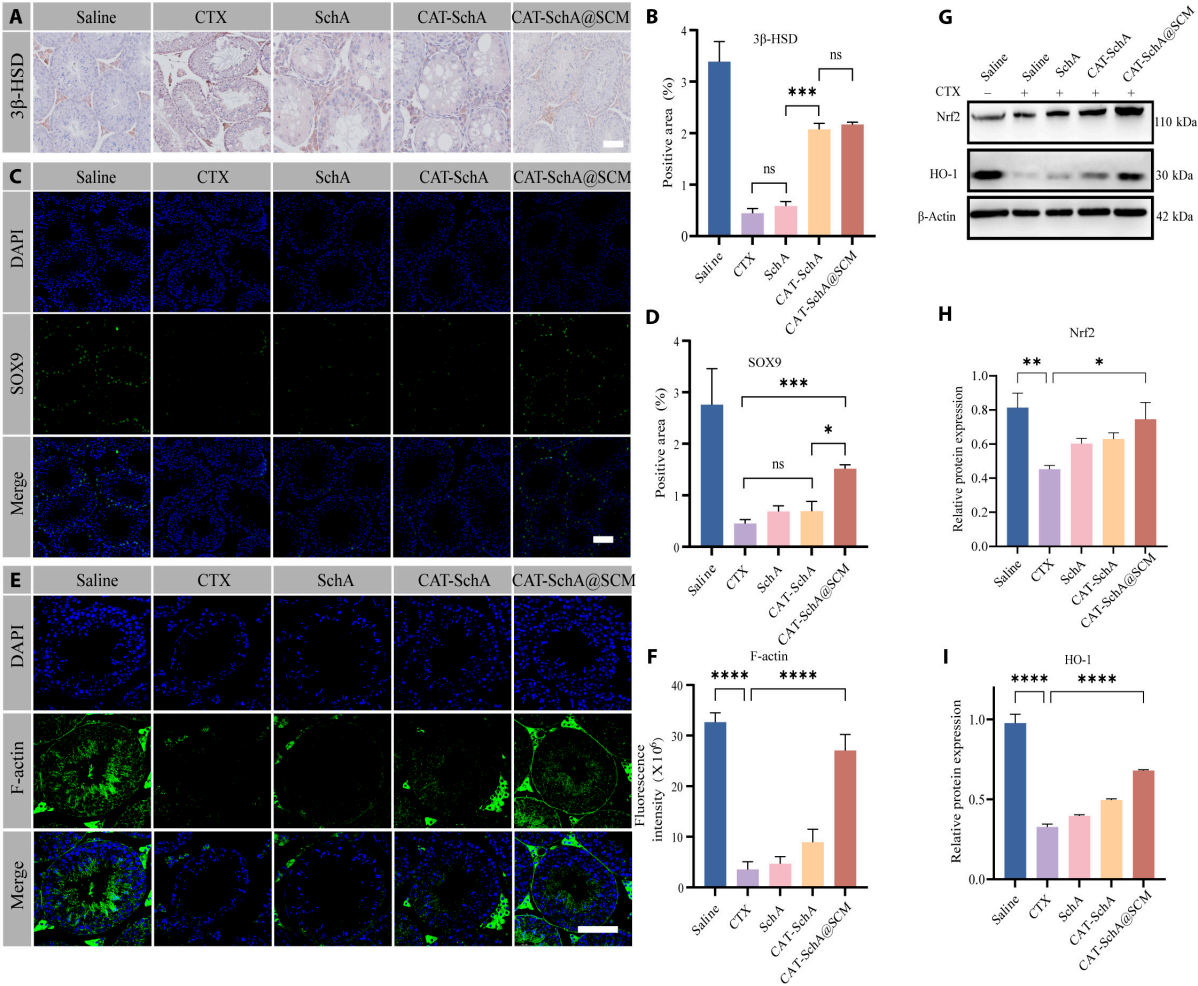
CAT-SchA@SCM improves CTX-induced testicular injury by regulating the Nrf2 and HO-1 pathways. (A) IHC detection of the expression of 3β-HSD protein in different groups. Scale bar, 100 μm. (B) Percentage of positive areas of 3β-HSD protein in different groups (*n* = 3). (C) IF detection of the expression of SOX9 protein in different groups. Scale bar, 100 μm. (D) Percentage of positive areas of SOX9 protein in different groups (*n* = 3). (E) IF detection of the expression of F-actin protein in different groups. Scale bar, 100 μm. (F) Fluorescence intensity quantification of F-actin protein in different groups (*n* = 3). (G) Western blot detection of Nrf2 and HO-1 proteins in different groups. (H) Quantification of the expression level of Nrf2 protein relative to β-actin in different groups (*n* = 3). (I) Quantification of the expression level of HO-1 protein relative to β-actin in different groups (*n* = 3). Data were presented as mean ± SEM. Statistical differences between 2 groups were analyzed by using 2-tailed Student’s *t* test. *P < 0.05, **P < 0.01, ***P < 0.001, and ****P < 0.0001.

To further explore the changes at the cellular level of the testis, we performed immunohistochemical (IHC) and IF staining to detect the markers of different types of testicular cells. 3β-HSD, the marker of Leydig cells, was used to exhibit the number of Leydig cells. It showed that the number of Leydig cells decreased significantly in the model group and recovered in both the CAT-SchA group and the CAT-SchA@SCM group (Fig. 6A and B). To detect the changes in the number of Sertoli cells, we performed IF staining of SOX9, the marker protein of Sertoli cells. Similar to the results of Leydig cells, the number of Sertoli cells decreased significantly in the model group. However, the recovery effect on the number of Sertoli cells was only observed to be significant in the CAT-SchA@SCM group and was significantly higher than that of the CAT-SchA group (Fig. 6C and D). In addition, we performed IF staining of F-actin, a protein complex localized in the acrosome of spermatids. The results showed that the number of spermatids was also decreased consistent with our hypothesis (Fig. 6E and F).

To explore the molecular mechanism of CAT-SchA@SCM, we verified the key antioxidant proteins Nrf2 and HO-1 by Western blot. The results showed that CAT-SchA@SCM could affect the Nrf2 signaling pathway, promote the nucleus translocation of Nrf2, and up-regulate the expression of HO-1, thereby playing a role in protecting Sertoli cells (Fig. 6G to I). These results fully demonstrated that CAT-SchA@SCM could rescue the testicular injury induced by CTX.

### Biosafety evaluation

**Fig. 7.**
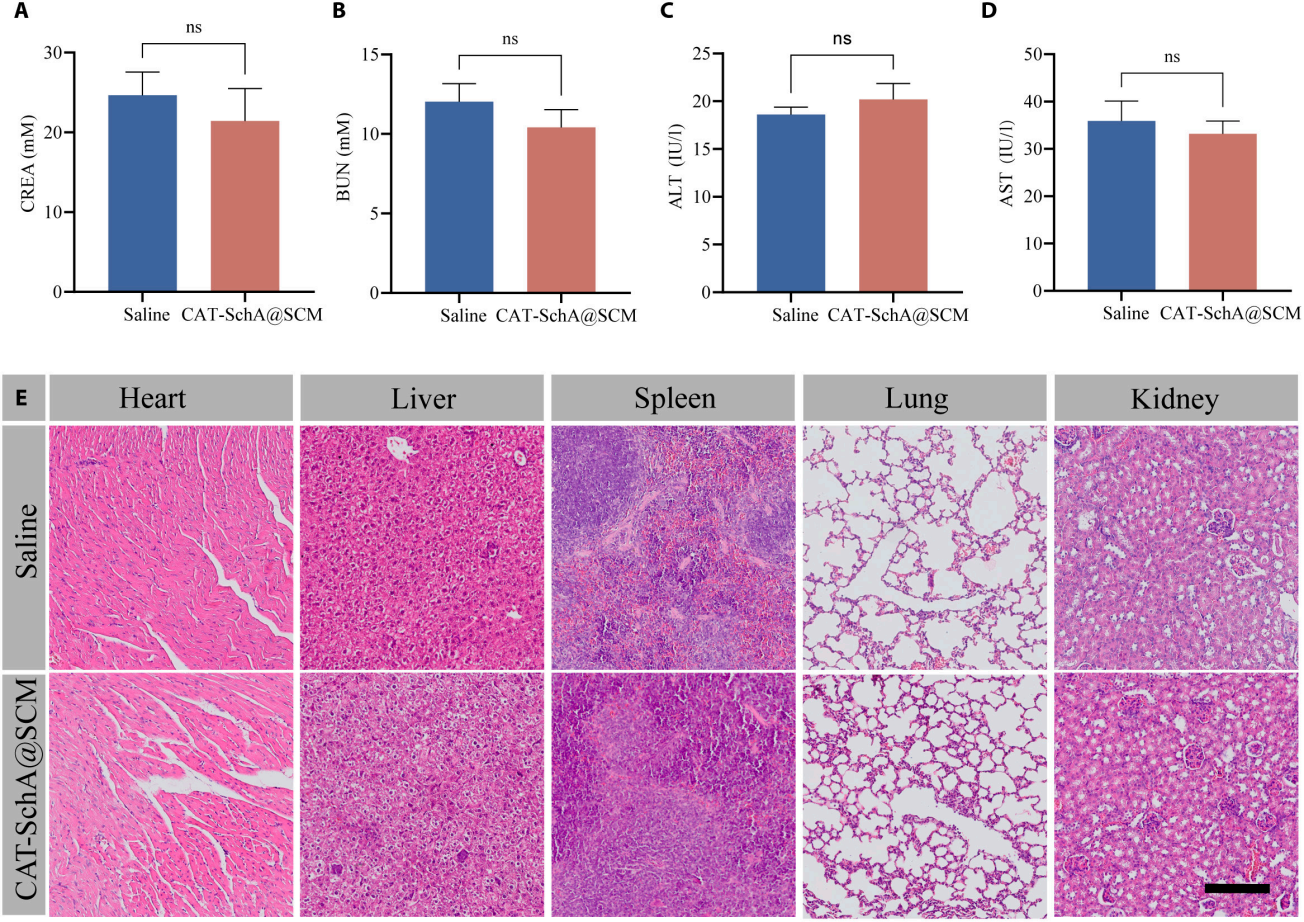
Biosafety of CAT-SchA@SCM. (A to D) Parameters of CREA (A), BUN (B), ALT aminotransferase (C), and AST aminotransferase (D) in normal mice after tail vein injection of CAT-SchA@SCM (*n* = 6). (E) H&E staining of major organs in normal mice after different treatments. Scale bar, 100 μm. Data were presented as mean ± SEM. Statistical differences between 2 groups were analyzed by using 2-tailed Student’s *t* test.

To determine the biosafety of the nanoparticles, we measured some serum biochemical indicators such as CREA, BUN, ALT, and AST, and no obvious abnormalities were found (Fig. 7A to D). In addition, we stained the main organs (including the heart, liver, spleen, lung, and kidney) of mice using the H&E staining method (Fig. 6E). The results showed that compared with the Saline group, no obvious atrophy, degeneration, or necrosis was observed in the pathological sections of the CAT-SchA@SCM tail vein group. In addition, in cell experiments, different concentrations of SchA, CAT-SchA, and CAT-SchA@SCM had no obvious cytotoxicity to TM3, TM4, and GC1 cells. Based on the above experimental results, the nanoparticles have good biosafety, which also implies their potential for clinical transformation.

## Conclusion

In summary, we developed a biomimetic nanoprotein delivery system (CAT-SchA@SCM). The goal was to endow the delivery system with certain tissue targeting properties through chemokines and adhesion molecules on the surface of homologous cells while enhancing the bioavailability and sustained-release characteristics of the drug. Animal experiment results showed that in a CTX-induced testicular injury model, the recovery effect of the CAT-SchA group was higher than that of the SchA group, indicating that encapsulation with the CAT protein carrier may improve the bioavailability of SchA and confer certain sustained-release capabilities. Additionally, the superior performance of the CAT-SchA@SCM group compared to the CAT-SchA group suggests that camouflaging CAT-SchA with biomimetic cell membranes enhances its stability and imparts testicular targeting properties.

Furthermore, small-animal imaging results demonstrated that CAT-SchA@SCM can target testicular tissue to a certain extent. We analyzed other major organs (Fig. S4A to F) and observed different metabolic behaviors between CAT-SchA@SCM and CAT-SchA@RBCM. Specifically, CAT-SchA@RBCM showed faster liver clearance, which may indicate that, compared with red blood cell membranes, Sertoli cell-derived coatings (as an immune-privileged cell source) prolong systemic circulation time and reduce liver-mediated rapid metabolism. Moreover, the prepared CAT-SchA@SCM exhibited good biocompatibility and high safety, effectively alleviating Sertoli cell apoptosis, up-regulating the expression of Nrf2 and HO-1, and thus ROS-related pathways.

In conclusion, this study not only provides a new strategy for treating CTX-induced testicular injury but also opens up new possibilities for treating oxidative stress-related diseases such as hypoxic injury, holding significant scientific and clinical application value.

## Data Availability

All data needed to evaluate the conclusions in the paper are present in the paper and/or the Supplementary Materials.
