# Supplementary material for: Testicular Sertoli Cell Membrane-Modified Schisandrin A-Loaded Catalase Nanoparticles Ameliorate Cytoxan-Induced Testicular Injury
Source: Biomater Res. 2025 Nov 4;29:0273. doi: 10.34133/bmr.0273 (PMC12583800; doi:10.34133/bmr.0273)
Supplement: Supplementary 1 — Figs. S1 to S4 [file bmr.0273.f1.docx]

**Testicular Sertoli Cell Membrane-Modified Schisandrin A-Loaded Catalase Nanoparticles Ameliorate Cytoxan-Induced Testicular Injury**

Yisong Ju^1#^, Li Lu^1,2,4#^, Jihai Liu^1#^, Lei Qian^1^, Haoqiang Zhang^1^, Fuying Zhu^4^,Yuhang Li^2^, Xun Wang^2^, Tao Song^1,2,4*^, Qingsong Ye^3*^, Ao Ma^1,2*^, Xiaozhi Zhao^1,2,3,4*^


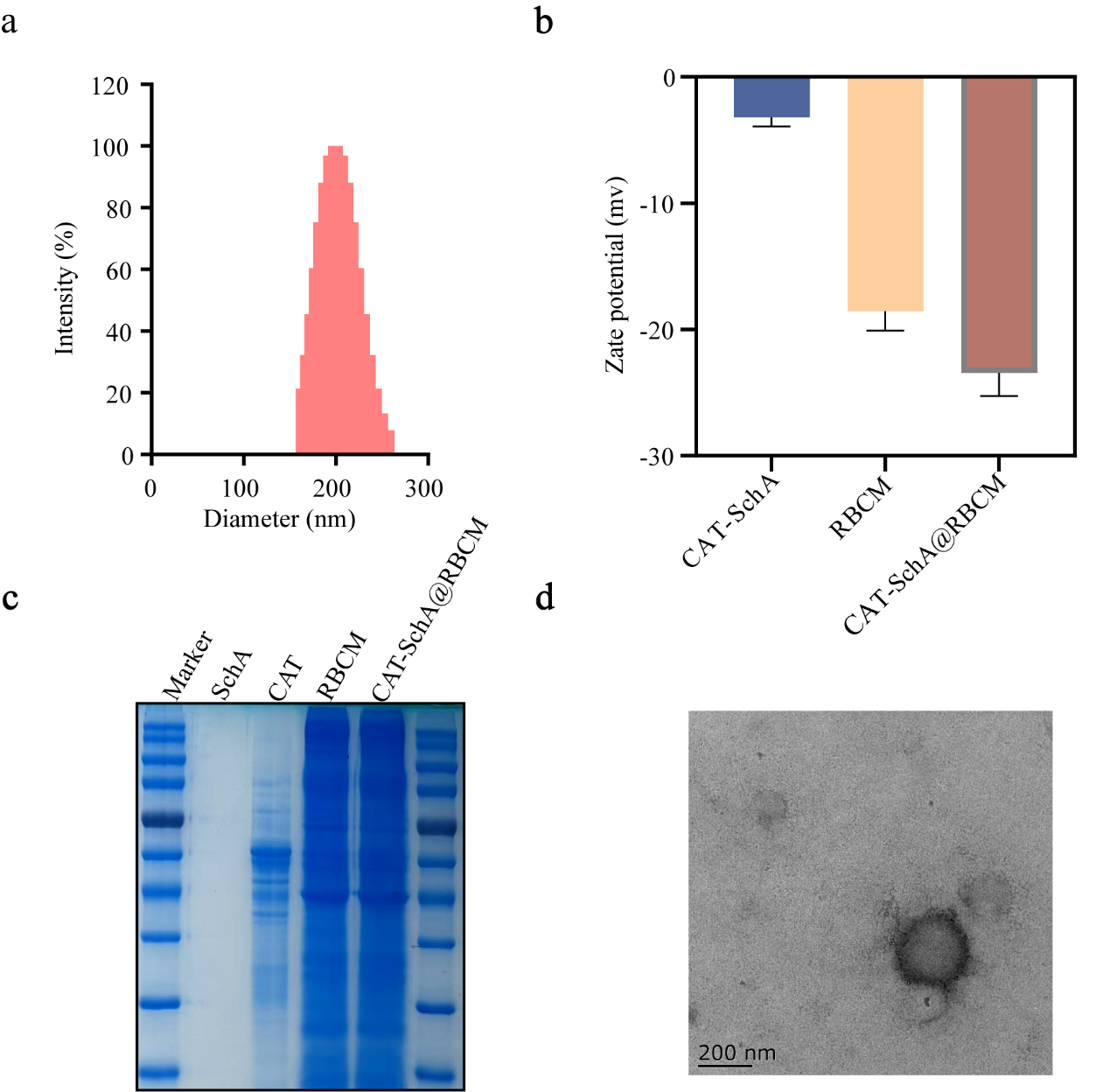


**Fig. S1 Preparation and Characterization of CAT-SchA@RBCM. (a)** Particle size distribution of the synthesized CAT-SchA@RBCM determined by Dynamic Light Scattering (DLS). **(b)** Zeta potentials of CAT-SchA and CAT-SchA@RBCM (n=3). **(c)** SDS-PAGE analysis of SchA, CAT, RBCM, and CAT-SchA@RBCM to determine the successful encapsulation of CAT-SchA by RBCM. **(d)** TEM image of CAT-SchA@RBCM. Scale bar: 200 nm.Values were presented as mean ±SEM.


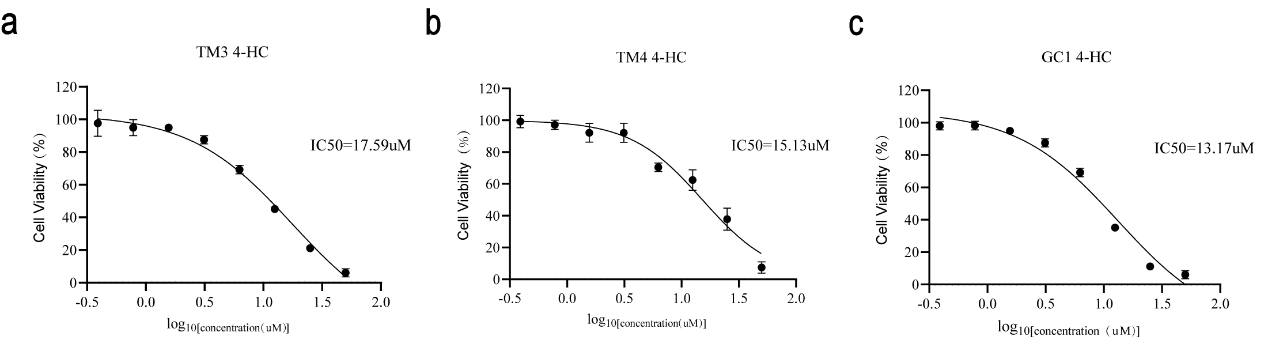


**Fig. S2 Exploration of the concentration of 4-HC causing cell damage. (a)** IC50 of 4-HC for the TM3 cell line (n=6) **(b)** IC50 of 4-HC for the TM4 cell line (n=6) **(c)** IC50 of 4-HC for the GC1 cell line (n=6).


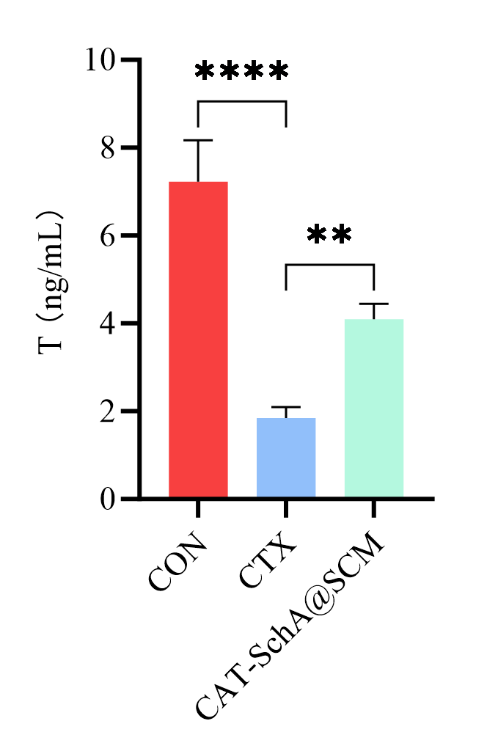


**Fig. S3** The index of serum testosterone in animal experiments (n=6). Date was presented as mean ± SEM. Statistical differences between two groups were analyzed by using two-tailed Student’s t-test. ns, no significant difference. **P < 0.01, ****P < 0.0001.

**
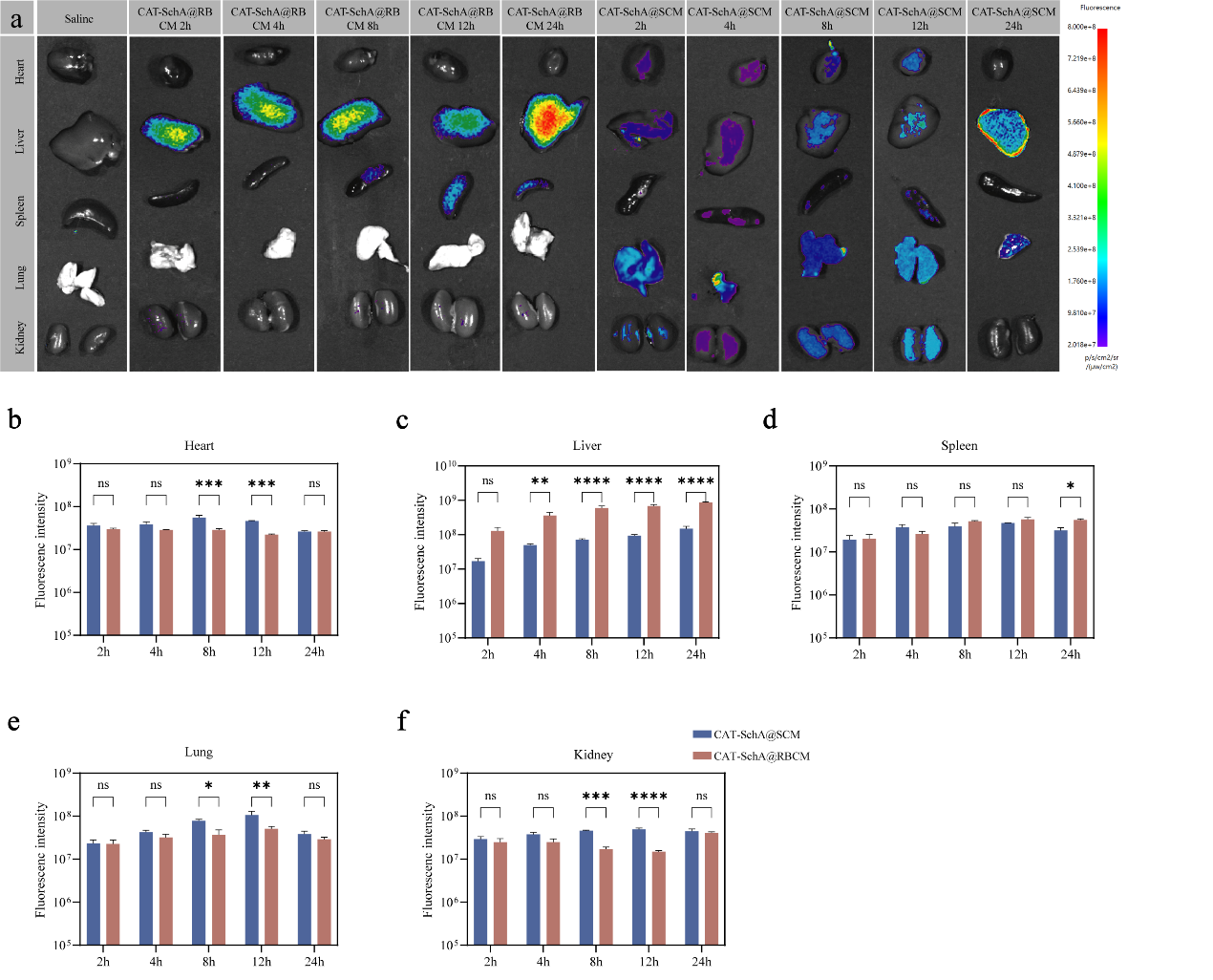
**

**Fig. S4 Biodistribution of CAT-SchA@SCM and CAT-SchA@RBCM in other internal organs in vivo. (a)** Ex vivo images of the heart, liver, spleen, lung, and kidney at different time points after intravenous injection of DiD-labeled CAT-SchA@SCM and CAT-SchA@RBCM **(b to f)** Quantitative analysis of the biodistribution of CAT-SchA@SCM and CAT-SchA@RBCM in major organs (heart, liver, spleen, lung, and kidney) after intravenous injection (n=3). Date was presented as mean ± SEM. Statistical differences between two groups were analyzed by using two-tailed Student’s t-test. ns, no significant difference. **P* < 0.05, ***P* < 0.01, ****P* < 0.001 and *****P* < 0.0001.
